# Bidirectional associations between mental health problems and language ability across 8 years of childhood

**DOI:** 10.1007/s00787-023-02192-x

**Published:** 2023-04-03

**Authors:** Nathalie Tamayo, Helen Wareham, Marie-Christine Franken, Cristina McKean, Henning Tiemeier, Pauline W. Jansen

**Affiliations:** 1 Department of Child and Adolescent Psychiatry/Psychology, Erasmus University Medical Center, Rotterdam, The Netherlands; 2 The Generation R Study Group, Erasmus University Medical Center, Rotterdam, The Netherlands; 3 School of Education, Communication and Language Sciences, Newcastle University, Newcastle Upon Tyne, UK; 4 Department of Otorhinolaryngology and Head and Neck Surgery, Erasmus University Medical Center, Rotterdam, the Netherlands; 5 Department of Social and Behavioral Science, Harvard T.H. Chan School of Public Health, Boston, USA; 6 Department of Psychology, Education and Child Studies, Erasmus University Rotterdam, Rotterdam, The Netherlands

**Keywords:** Children, Development, Internalizing symptoms, Externalizing symptoms, Language ability

## Abstract

Research examining the development of behavior, emotions and language, and their intertwining is limited as only few studies had a longitudinal design, mostly with a short follow-up period. Moreover, most studies did not evaluate whether internalizing symptoms and externalizing symptoms are independently associated with language ability. This study examines bidirectional associations between internalizing symptoms, externalizing symptoms and language ability in childhood in a large, population-based cohort. Longitudinal data from the Millennium Cohort Study, a cohort of children in the United Kingdom followed from birth to 11 years (*n* = 10,878; 50.7% boys), were analyzed. Internalizing and externalizing symptoms were based on parent reports. Language ability (higher scores reflecting poorer ability) was assessed by trained interviewers at ages 3, 5, 7 and 11 years. Structural Equation Models (SEM) were performed, including random-intercept cross-lagged panel models (RI-CLPM) and cross-lagged panel models (CLPM). Internalizing symptoms, externalizing symptoms and language ability were stable over time and co-occur with each other from early life onwards. Over time, externalizing symptoms in early childhood were associated with less growth in language skills and with increases in internalizing symptoms. In late childhood, language ability was negatively associated with later internalizing and externalizing symptoms. The early start, co-occurrence and persistent nature of internalizing symptoms, externalizing symptoms and (poorer) language ability highlights the importance of comprehensive assessments in young children who present problems in one of these domains. Specifically, among children in the early grades of elementary school, those with language difficulties may benefit from careful monitoring as they are more likely to develop difficulties in behavior and emotions.

**Supplementary Information:**

The online version contains supplementary material available at 10.1007/s00787-023-02192-x.

## Introduction

Child development describes the process of changes from infancy to adulthood, incorporating physical growth, cognitive (e.g. intellectual and language) and social-emotional development. These domains are intertwined and the current study is focused on how language and socio-emotional development influence each other. Important indicators of socio-emotional development are behavior and emotions, which is broadly categorized as internalizing symptoms including anxiety and depressive symptoms, and externalizing symptoms including aggressive behavior and attention difficulties. Language ability refers to multiple language-related domains reflecting receptive language, which denotes the ability to comprehend language, and expressive language, which encompasses the capacity to produce language [1].

Describing emotional and behavioral problems in very young children can be challenging, as poor control of emotions and defiant behaviors can be indicative of problems but are also part of normative development in early childhood. Evidence shows that children as young as 1.5 years show internalizing symptoms as emotional reactivity, somatic complaints, anxiety and depression symptoms, withdrawn symptoms or peer difficulties, with considerable variation in symptoms across children. Externalizing symptoms at a young age are presented as aggression, inattention and hyperactivity [2, 3].

In the general population, developmental trajectories of internalizing and externalizing symptoms and language ability tend to be relatively stable from childhood to adolescence. On the one hand, the large majority of children follow a trajectory that is characterized by a consistent absence or very low level of symptoms (e.g., no internalizing symptoms for 64% and no externalizing symptoms for 82% of children followed from ages 7 to 15 [4]; and stable, adequate language ability for 94% of children followed from ages 4 to 11 [5]). On the other hand, of the children whose trajectories do describe changes in symptoms, almost all displayed a downward trajectory of increasing internalizing and externalizing symptoms or less growth in language skills with increasing age [4, 5].

From an early age onwards, internalizing symptoms, externalizing symptoms and language ability difficulties can co-occur. For instance, in a Dutch population-based study among 3 year-olds, 13.5% of the children presented with internalizing and externalizing problems, of whom 2% showed a profile of co-occurring symptoms [6]. Likewise, in a group of 418 children aged between 2 and 5 years, a negative association of verbal ability with internalizing and externalizing symptoms has been found [7]. Moreover, studies indicate that children with symptoms in one domain are more likely to develop symptoms in another domains over the next years. In the same Dutch cohort, 1.5 year-olds’ lower expressive language ability was associated with more internalizing symptoms at age 3 [8]. Likewise, poorer expressive language ability at age 3 was related to the development of externalizing symptoms at age 5, even after adjusting for parenting practices [9]. Another study showed that poorer receptive language abilities in 4–7 year old children were associated with higher internalizing symptoms and externalizing symptoms from ages 8 to 15 [10]. Research examining the opposite direction showed that internalizing symptoms were negatively related to language expressive skills [11], while externalizing symptoms were negatively associated with receptive, but not expressive language skills [12]. Together, these findings suggest a bidirectional relationship between internalizing symptoms, externalizing symptoms and language ability, although it should be noted that the explained variance in most studies was low (from 0.03–3%) in the two studies that reported on the explained variance [7, 8]. However, given that studies mostly focused on early childhood, had a cross-sectional design or had a short follow-up [6–12], it is unclear how these relationships further take shape across childhood.

In sum, evidence suggests that children's internalizing symptoms, externalizing symptoms and language development (1) mostly follow a stable trajectory, and (2) tend to co-occur, with (3) the presence of symptoms in one domain being bidirectionally associated with the development of symptoms in other domains. Yet, current research is limited in three important ways. First, the few population-based, longitudinal studies do not have long follow-up periods, limiting knowledge about longer term, and potentially changing associations. Second, no studies to date have considered the trait-like nature, i.e. temporal stability, of internalizing symptoms, externalizing symptoms and language ability [9, 11, 12], which requires a specific analytic approach that accounts for the stability of the constructs over time [13]. Therefore, previous estimates of the bidirectional associations between internalizing symptoms, externalizing symptoms and language ability may have been overestimated. Third, most studies did not evaluate internalizing symptoms, externalizing symptoms and language ability in an integrated manner [4–6, 8, 12, 14], which might lead to biased results. For example, internalizing symptoms and externalizing symptoms both negatively related to language ability [11, 12]. However, it is not known if associations are driven by the presence of the other symptoms (because they often co-occur) or if internalizing and externalizing symptoms are independently associated with language ability. To address these limitations, we examined the development of internalizing symptoms, externalizing symptoms and language ability in an integrated model to determine the stability and direction of their effects from ages 3 to 11 years in a large population-based cohort. The large dataset allows us to thoroughly adjust for several covariates that may confound the associations. We hypothesized weaker associations between internalizing symptoms, externalizing symptoms and language ability than previously reported, due to accounting for the co-occurrence between internalizing symptoms and externalizing symptoms and the trait-like nature of internalizing symptoms, externalizing symptoms and language ability.

## Method

### Participants

Participants were drawn from the Millennium Cohort Study (MCS), a population-based prospective cohort that enrolled 19,483 children from born in the UK in 2000 and 2001. The MCS sample is representative of the total UK population when weighted. Families living in disadvantaged areas, those of ethnic minority backgrounds in England and those in the smaller nations of the UK were over-sampled. There have been 7 sweeps of data collection [15], of which the sweeps at child ages 3, 5, 7 and 11 years included relevant data on internalizing symptoms, externalizing symptoms and language. Ethical approval for the MCS was gained from the NHS Multi-Centre Ethics Committee. Parents and, from age 11 onwards, children gave informed consent before interviews took place. Only the first child registered within a household was included in our study (*n* = 252 excluded). Furthermore, children were excluded if the main language spoken at home was not English (*n* = 3064 excluded) as these children had lower scores on the language assessments (data not shown), and if they did not have at least one assessment of each concept (internalizing symptoms, externalizing symptoms and language ability score, *n* = 1793 excluded). This resulted in a study population consisting of 14,464 children for the present study.

### Internalizing and externalizing symptoms

Internalizing and externalizing symptoms were assessed by parental reports (> 98.5% mother report) at ages 3, 5 and 7 years and by teacher report at 7 and 11 years. For this, the Strengths and Difficulties Questionnaire (SDQ) was used. The SDQ is a widely used inventory that is a reliable and validated screening tool to measure emotional and behavioral difficulties [3]. The factor structure has been shown acceptable in a previous study, while the internalizing-externalizing symptoms contrast showed a satisfactory convergent and discriminant validity [16]. Moreover, predictive validity has been shown by internalizing and externalizing symptoms in early childhood predicting internalizing symptoms at later ages [17]. The SDQ includes five subscales: emotional symptoms, conduct problems, hyperactivity, peer problems and prosocial behavior. In line with recommended practice two composite scales were used [16]. The externalizing problem scale comprises the 10 items from the conduct problems and hyperactivity subscales (e.g. “Often has temper tantrums or hot tempers”). The internalizing problem scale comprises 10 items from the emotional symptoms and peer problems subscales (e.g. “Generally liked by other children”). Items were scored on a three-point scale from “not true” (0) to “certainly true” (2) and were summed. The sum scores on these two composite scales have a range of 0–20 with higher scores indicating more symptoms. The internal consistency (Cronbach’s alpha) for the two composite scales ranges from 0.61 (internalizing at age 3) to 0.81 (externalizing at age 11) in this cohort [18]. As the internal consistencies were quite low for some of the scales, we evaluated the factor structure for the internalizing and externalizing scales at all ages. A good factor structure was confirmed for each scale at each age by good model fit indices as presented in Supplementary Table 1 (cut off points of good model fit are described in Statistical Analysis section). For example at age 3, the internalizing symptoms construct showed a satisfactory Root Mean Square Error of Approximation (rRMSEA) = 0.04, Standardized Root Mean Square Residual (SRMR) = 0.02 and Comparative Fit Index (CFI0) = 0.95. A description of the reporter of behavior, emotions and language, as well as the age at assessments is presented in Supplementary Table 2.

### Language assessments

Expressive vocabulary was assessed by a trained interviewer with scales from The British Ability Scales Second Edition (BAS II). The BAS II has demonstrated construct validity as a measure of cognitive ability and has a high test–retest reliability [19]. The individual scales of the BAS II yield robust and individually interpretable sub-tests [20]. The BAS II scales incorporate items of increasing difficulty depending on a child’s age, and initial result adaptations are made. For example, if the child gives a wrong answer on an initial item, the child is presented an easier item. Halfway through the test there is a decision point to either continue or end the test depending on the child’s performance [21]. This means that children complete different (numbers of) test items. Therefore, the test items have been analyzed using the Rasch model of item response theory to develop a conversion of the child’s raw score, based on the items they answered, so that scores are comparable between children. In the current study, the following individual scales were used: naming vocabulary, reading, and verbal similarities. At ages 3 and 5 years, the naming vocabulary scale was used [22]. The child was shown a set of pictures with increasing difficulty and had to name them (e.g. Scissors, Window, Brush, Watch, etc.), with the starting picture depending on the age of the child. At age 7, the BAS II word reading test was administered. The child read aloud a series of words with increasing difficulty that are presented on a card (e.g. The, Up, He, You, etc.). At age 11, performance on the BAS II verbal similarities was assessed. The child was given three stimulus words and asked to name the class to which all the examples belong (e.g. Banana, Apple, Orange). The reading assessment at age 7 differs from the language assessments at ages 3, 5 and 11, which are semantic tests. The cross-sectional correlation between the reading and semantic tests is moderate (0.3 to 0.4) and these tests are associated to one another longitudinally (0.3 to 0.7) [23, 24]. In the present study, the correlation between the different language assessments ranges from 0.33 between ages 3 and 11 to 0.40 between ages 3 and 5 (Supplementary Table 3). All assessment scores were standardized for child age [25] and were further standardized to a mean of 10 (SD = 5), with lower scores indicating poorer language ability, i.e. more language difficulties.

### Covariates

We controlled for several time-invariant covariates, including child sex and ethnicity categorized as white or other, in accordance with guidelines from the Office for National Statistics [26]. Mother’s age at birth and mother’s education (categorized as up to secondary schooling, vocational qualification, and tertiary education) were also considered covariates. Furthermore, income was scored as a modified OECD equivalization where each scale sets the family need relative to those with couples with no children [25]. Single parenthood was measured in the first wave or the second in case of missing data in the first [25]. Single parenthood was categorized into a dichotomous variable indicating if the child lived with one parent or with two parents (both biological or adoptive parents; or a biological parent and a step parent). Additionally, we controlled for child non-verbal IQ, calculated as the principal component from the BAS II picture similarities and BAS II pattern construction assessed at age 5 years. Picture similarities assesses non-verbal problem solving (inductive reasoning) and visual perception skills. Pattern construction assesses cognitive processes related to visual/spatial analysis, visual/spatial matching and problem solving including the ability to synthesize and use strategies. These individual scales are core scales that form the non-verbal component of the BAS II estimate of intelligence [20]. Finally, harsh parental discipline was evaluated with a parent report of the Conflict Tactics Scale at age 3 [27]. A sum score was created and the Cronbach’s alpha in the current sample was 0.70.

### Statistical analysis

**Fig. 1:**
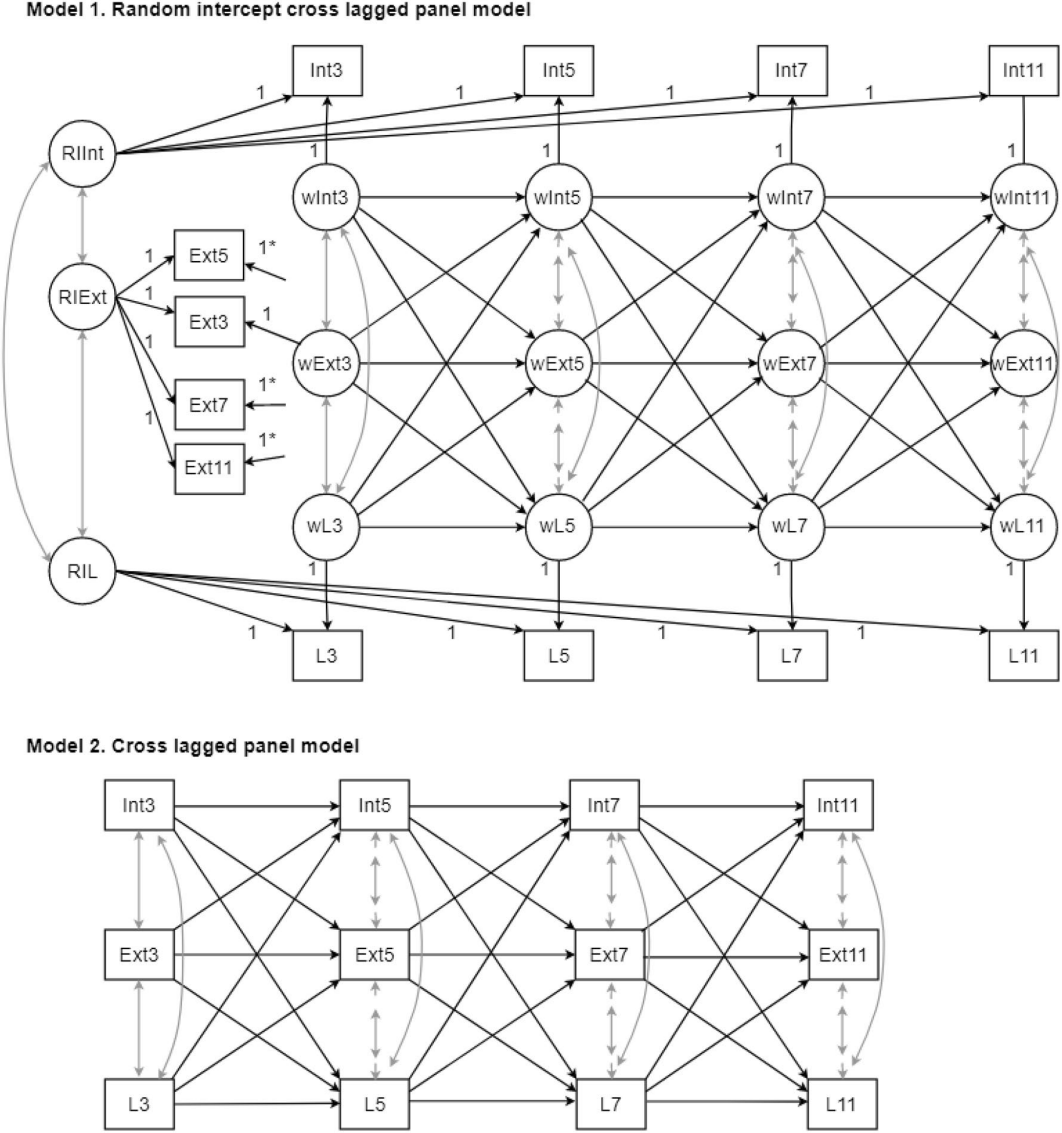
Outline of the models. Prefixes: *W* latent variable. *RI* random intercept. *Int, Ext and L* Internalizing and externalizing problems and language assessments. Suffixes 3, 5, 7 and 11: represent age at assessment. Gray arrows represent variances, covariances and residual covariances. *Arrows should be connected to their latent variable as “Ext3”and “wExt3” are. These arrows are not connected to their latent variable for simplification

Structural equation models (SEM) fitted in lavaan (version 0.6-7) with R (version 4.04) were used to evaluate bidirectional associations between internalizing symptoms, externalizing symptoms and language ability. We first evaluated if the constructs had a time-invariant nature, as bias may arise if constructs have a time-invariant nature and this is not accounted for. Two models were fitted: a random-intercept cross-lagged panel model (RI-CLPM) accounting for the time-invariant nature of the three constructs (Fig. 1, Model 1), and a cross-lagged panel model CLPM that did not account for this (Fig. 1, Model 2). The two models were compared as suggested by Hamaker et al. [28]. The RI-CLPM assesses change over time as deviations from the invariant nature of the constructs. To do so, the RI-CLPM has three components [28]: (1) the grand means, which are the means of internalizing and externalizing symptoms and language assessments per occasion; (2) the between components which are the random intercepts of internalizing and externalizing symptoms and language assessments that capture a subject’s time-invariant deviation from the grand means and thus represent the stable differences between constructs; (3) the within components that reflect differences between a subject’s observed measurements and the subject’s expected score based on the grand means and its random intercepts. On the other hand, the CLPM only allows to control for the stability of constructs over time through the inclusion of auto-regressive relationships. Nonetheless, if the stability of the constructs is trait-like with a time-invariant nature, the inclusion of only auto-regressive parameters will fail to adequately control for this invariance and results might be biased [13]. The two models were compared based on a *χ*^2^ difference test [28] and model fit indices were inspected, including the robust Comparative Fit Index (CFIr; acceptable fit ≥ 0.90), the robust Root Mean Square Error of Approximation (RMSEAr; acceptable fit ≤ 0.06) and the Standardized Root Mean Square Residual (SRMR; acceptable fit ≤ 0.08) [29]. For all models, standardized coefficients are presented, representing SD change in the outcome per 1 SD change in the exposure.

We performed three sensitivity analyses. First, considering the evidence regarding sex differences in internalizing and externalizing symptoms, and language ability, we performed a stratified analysis by child sex [8]. Second, we additionally adjusted the models for child non-verbal IQ to rule out possible confounding by child intelligence. Third, we replaced the mother reported internalizing and externalizing symptoms at age 7 years by the teacher rating to assess the consistency of results across different informants. All analyses included the covariates listed above. Survey weights were applied to account for the stratified cluster sample design. Missing values were imputed by multiple imputation using chained equations, with package mice [30]. About 32.6% of the children had missing data, with 70.1% of them missing data in less than 20.0% of the variables. Twenty imputed data sets were created and the presented results are the estimates averaged across these twenty data sets.

## Results

The characteristics of the children included in this analysis are presented in Table 1. There was a similar proportion of boys (51.2%) and girls, with the majority being white (93.6%). The mean internalizing scores were similar from ages 3 to 11 (all around 2.6), while the externalizing scores decreased, from a mean score of 6.7 at age 3 to a mean score of 2.9 at age 11. It was not possible to determine age trends in language scores due to differences in the assessment instruments. Mean maternal age at child birth was 24.5 years. One third of the mothers (32.2%) had a tertiary education, 15.3% had achieved a vocational qualification and had (52.5%) had less than vocational qualification.

**Table 1:** Characteristics of the Study Population

	School achievement
*Characteristics of the Child*	
Sex, boy, %	51.2
Child race, %	
White	93.63
Other	6.37
At 3 years assessment	
Child age (in years), Mean (SD)	3.1 (0.2)
Internalizing problem score, Mean (SD)	2.9 (2.5)
Externalizing problem score, Mean (SD)	6.7 (3.8)
Naming vocabulary score, Mean (SD)*	50.5 (15.9)
At 5 years assessment	
Child age (in years), Mean (SD)	5.2 (0.2)
Internalizing problem score, Mean (SD)	2.5 (2.4)
Externalizing problem score, Mean (SD)	4.9 (3.4)
Naming vocabulary score, Mean (SD)*	54.7 (17.2)
Pattern construction, Mean (SD)	49.9 (10.8)
Pictures similarities at five years, Mean (SD)	55.2 (11.0)
At 7 years assessment	
Child age (in years), Mean (SD)	7.2 (0.2)
Internalizing problem score, Mean (SD)	2.7 (2.7)
Externalizing problem score, Mean (SD)	4.7 (3.6)
Word reading score, Mean (SD)*	110.4 (30.3)
At 11 years assessment	
Child age (in years), Mean (SD)	10.7 (0.5)
Internalizing problem score, Mean (SD)	2.7 (3.0)
Externalizing problem score, Mean (SD)	2.9 (3.5)
Verbal similarities score, Mean (SD)*	58.7 (16.2)
*Characteristics of the mother*	
Mother’s age at intake (in years), Mean (SD)	24.5 (6.0)
Maternal education, %	
Up to secondary schooling	52.5
Vocational qualification	15.3
Tertiary education	32.2
*Characteristics of the family*	
Single parenthood, %	17.9
Parenting at age 3, Mean (SD)	13.0 (5.0)
Income, Mead (SD)	305.6 (199.6)

*N* 14,463, *SD* Standard deviation

*Unstandardized values

### Bidirectional models of internalizing and externalizing symptoms and language ability

**Fig. 2:**
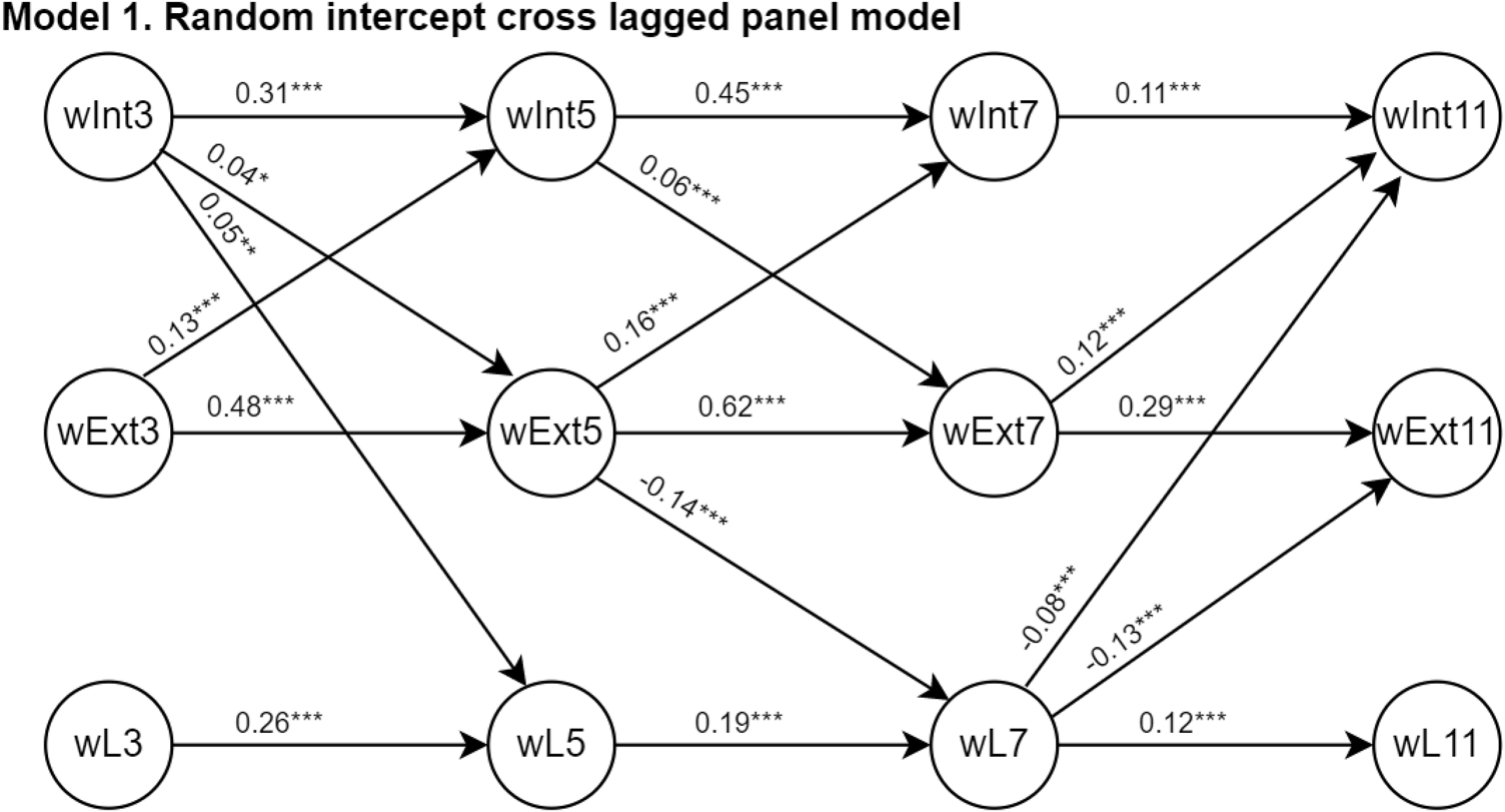
Bidirectional association of internalizing and externalizing problems and language ability. The output corresponds to the RI-CLPM’s auto-regressive and cross-lagged paths depicted as Model 1 in Supplementary Fig. 1. The complete output of all these paths is presented in Supplementary Table 4. Prefixes: *W* latent variable. *Int, Ext and L* Internalizing and externalizing problems and language assessments. Suffixes 3, 5, 7 and 11: represent age at assessment

The χ^2^ difference test comparing RI-CLPM and CLPM (value = 2292, p < 0.001, degrees of freedom = 90 and 84, respectively) suggested a better fit for the data with the RI-CLPM. This was confirmed by the other model fit indicators, with the RI-CLPM having a better model fit (CFIr = 0.94; RMSEAr = 0.06; SRMR = 0.04) than the CLPM (CFIr = 0.91; RMSEAr = 0.07; SRMR = 0.05). Consequently, we focus on the results of the RI-CLPM´s auto-regressive and cross-lagged paths. The significant auto-regressive and cross-lagged paths are presented in Fig. 2 and the complete output of the paths is presented in Supplementary Table 4. For comparison, the results of the CLPM are also presented (Model 2 in Supplementary Fig. 1 and Supplementary Table 4). The CLPM model, that did not account for the trait-like nature of internalizing and externalizing symptoms and language ability, yielded 22 significant paths, while the RI-CLPM yielded 18 significant paths. For instance, of the three possible longitudinal associations between externalizing scores and expressive vocabulary (ages 3 to 5; 5 to 7; and 7 to 11), the CLPM had three statistically significant associations whereas the RI-CLPM only had one, from age 5 to 7.

For the RI-CLPM, all auto-regressive paths of internalizing scores, externalizing scores and language ability showed consistent associations between wave-on-wave assessments. For example, per one SD higher internalizing score at age 3, the internalizing score at age 5 increased 0.31 (95% CI [0.27, 0.35] *p* < 0.001). Similar wave-on-wave associations between internalizing scores over time were seen from age 5 to 7 (Beta = 0.45, 95% CI [0.41, 0.49] *p* < 0.001) and from age 7 to 11 years (Beta = 0.11, 95% CI [0.05, 0.13] *p* < 0.001). The internalizing and externalizing scores were also associated with each other over time. For example, the externalizing problem score at age 3 was positively associated with the internalizing score at age 5 (Beta = 0.13 SD, 95% CI [0.10, 0.16] *p* < 0.001). The only exception was that the internalizing score at age 7 did not relate to the externalizing score at age 11.

Next, we describe the cross-lagged paths between the internalizing or externalizing scores and language ability scores, which denote any bidirectional associations between these constructs. Only two paths were significant: (1) the internalizing score at age 3 was positively associated with the language ability score at age 5 (Beta = 0.05 SD, 95% CI [0.02, 0.07] *p* = 0.002), and (2) the externalizing score at age 5 was negatively related to language ability at age 7 (Beta = − 0.14 SD, 95% CI [− 0.17, − 0.12] *p* < 0.001). Finally, regarding the association between language ability and internalizing or externalizing scores: the language ability score at age 7 was negatively associated with the internalizing score (Beta = − 0.08 SD, 95% CI [− 0.12, 0.06] *p* < 0.001) and negatively associated with externalizing score (Beta = − 0.13 SD, 95% CI [− 0.17, 0.10] *p* < 0.001) at age 11 years.

### Sensitivity analysis

**Fig. 3:**
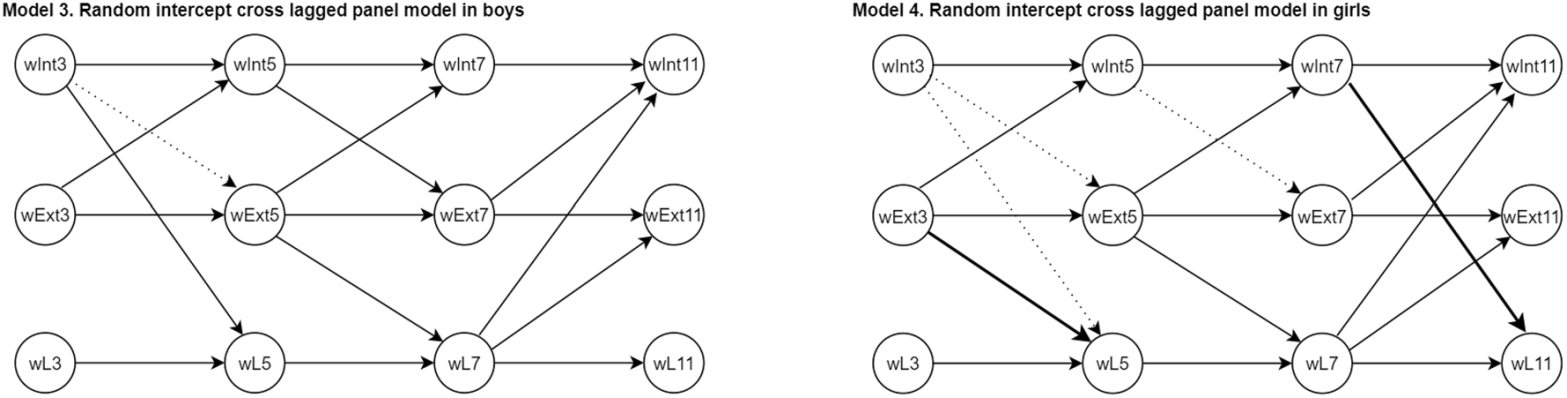
RI-CLPM of the bidirectional association of internalizing and externalizing problems and language ability stratified for boys and girls. Each model corresponds to the RI-CLPM’s auto-regressive and cross-lagged paths. The complete output of all these paths is presented in Supplementary Table 5. Prefixes: *W* latent variable. *Int, Ext and L* Internalizing and externalizing problems and language assessments. Suffixes 3, 5, 7 and 11: represent age at assessment. Dotted arrows represent non-significant paths in models 3 and 4 that were significant in the RI-CLPM presented in model 1. Bold arrows represent a significant paths in model 4 that were non-significant in the RI-CLPM presented in model 1

First, we performed a stratified analysis for boys and girls. The stratified results for boys were largely similar to the non-stratified analysis (comparison of Model 3 in Fig. 3 with Model 1 in Fig. 2, and in Supplementary Table 5), the only difference was that internalizing problems at age 3 years were not associated with the externalizing problems scale at age 5. For girls results changed in five paths. Although the effect estimates were fairly similar to the results in the complete sample, the statistical significance in some of the paths changed (comparison of Model 4 in Fig. 3 with Model 1 in Fig. 2, and in Supplementary Table 5). Several associations (internalizing symptoms at age 3 with language ability score at age 5; internalizing symptoms at ages 3 and 5 with externalizing symptoms at ages 5 and 7) were no longer statistically significant. On the other hand, the non-significant associations between externalizing symptoms at age 3 and the language ability score at age 5, and between internalizing symptoms at age 7 and language ability score at age 11, were significant in girls. Second, child intelligence can be a confounder of the association between internalizing (or externalizing) scores and language ability, and therefore, we additionally adjusted the models for child IQ. Results remained similar to the main analyses (Model 5 in Supplementary Fig. 2 and Supplementary Table 6). Finally, when replacing the mother report on internalizing and externalizing problem scores at age 7 by teacher report (Model 6 in Supplementary Fig. 2 and Supplementary Table 6), two associations were no longer significant (i.e. cross-lagged associations of internalizing problem scores at ages 3 and 5 years with externalizing problem scores at ages 5 and 7, respectively). The other associations remained significant and did not substantially change.

## Discussion

A nationally-representative, prospective cohort of children was used to study the bidirectional relationships between internalizing symptoms, externalizing symptoms and language ability from ages 3 to 11 years. Our findings support (1) the early start, and the co-occurrence and persistent nature of internalizing symptoms, externalizing symptoms and language ability difficulties; (2) above the co-occurrence and early start of symptoms, externalizing symptoms related positively to internalizing symptoms at later ages while the association of internalizing symptoms with externalizing symptoms was less consistent; and (3) a less consistent longitudinal association of internalizing and externalizing symptoms with language ability, reflecting small effects when present.

Internalizing symptoms, externalizing symptoms and poor language ability first appeared early in life, co-occurred and were persistent throughout childhood, as demonstrated by the RI-CLPM having a better model fit than the CLPM. The random intercepts of the RI-CLPM represent the early appearance and stability of internalizing symptoms, externalizing symptoms and language ability. The correlation structure between the random intercepts of the RI-CLPM represents their co-occurrence. Not accounting for the time-invariant nature of internalizing and externalizing symptoms and language ability and their correlation, as shown by the CLPM results, leads to more significant associations when assessing their relation. The early appearance and co-occurrence of internalizing symptoms, externalizing symptoms and language ability may reflect the presence of common risk factors for the development of internalizing and externalizing symptoms and language ability early in life. Indeed, internalizing symptoms and externalizing symptoms seem to share some genetic etiology [31], that can reflect shared biological mechanisms, for instance delayed emotional maturity, albeit the expression of symptoms may be at different time points. Furthermore, perinatal complications and preterm birth have been associated with both externalizing symptoms and language difficulties [32, 33], and internalizing symptoms and developmental language disorder also have been shown to have shared genetic risk factors [34]. The stability of internalizing symptoms, externalizing symptoms and language ability is in line with previous studies showing that during childhood, the majority of children follow persistent trajectories of no or few internalizing symptoms and no or few externalizing symptoms [4], and a consistent developmental trajectory of language abilities [5] up to adolescence.

In addition, we found that externalizing symptoms consistently and positively predicted the development of internalizing symptoms. This finding is consistent with previous literature studying the joint development between externalizing and internalizing symptoms. Six-year-old children with high externalizing scores had a 40% probability of developing internalizing symptoms up to age 9 [14]. The positive longitudinal association of externalizing symptoms and internalizing symptoms has further been explored by Gooren’s et al*.* in 5-year-olds in whom peer rejection mediated the association between externalizing symptoms and internalizing symptoms over a two year period [35]. The longitudinal positive association of internalizing symptoms with externalizing symptoms was less strong and did not sustain in sensitivity analyses by sex or when teachers reported symptoms. These findings suggest that large sample sizes are needed to detect small differences that are less evident when examining boys and girls separately or that might not be picked up by teachers who generally underreport internalizing symptoms as compared to children or their parents [36, 37].

Regarding the development of internalizing symptoms, externalizing symptoms and language ability, our results support a bidirectional association between behavior, emotions and language ability. Unexpectedly, we found a small, positive association of internalizing symptoms at age 3 with language ability at age 5. In the sensitivity analysis, this positive association was statistically significant only for boys, however, girls had a similar, yet non-significant estimate. Therefore, we interpret these results as suggestive of no sex interaction. A positive longitudinal association between internalizing symptoms and better school achievement when accounting for the co-occurrence with externalizing symptoms has been noted in two different Dutch cohorts [38, 39] and a Finish cohort [40]. Although the mechanisms behind this result have not been explored, we hypothesize that in the absence of attention problems, some anxiety may motivate children to work harder on learning activities and may lead to a better cognitive performance. As shown in a study among adolescents with ADHD, those who reported mild anxiety performed better in cognitive tasks as compared to peers without ADHD [41]. Alternatively, the unexpected association may be partly explained by the language catch-up of late talkers, who are often characterized as ‘inhibited’, a concept which overlaps with internalizing problems and thus may be reflected in its reports [42].

Besides, we found a small negative association between child externalizing symptoms at age 5 and expressive language ability at age 7. This is in contrast to previous research by Gigi et al. showing that externalizing symptoms were negatively related to receptive language but not to expressive language [12]. The differences in results could be explained by study characteristics. In the present study children came from white families who spoke English and had mostly high educated mothers, whereas Gigi et al. studied children from ethnic minorities living in poverty [12]. We hypothesize that social conditions may play a role in the effect externalizing symptoms have on language development. Regardless, both study results support the hypothesis that externalizing symptoms might limit children’s capacity to acquire language abilities [43]. The negative effects of externalizing symptoms, especially the hyperactivity and inattention domains, have been noted for multiple child developmental outcomes like child non-verbal intelligence [44] and school achievement [38]. Hyperactivity and attention problems can limit the ability to choose and concentrate on relevant stimuli, interfering with the learning process affecting cognitive development, including language, and academic competencies.

Additionally, we found a small effect of expressive language ability at age 7 being negatively related to internalizing and externalizing symptoms at age 11. This results is consistent with evidence relating language ability to internalizing and externalizing symptoms [45]. These findings may be explained by the hypothesis that children with more advanced language abilities can better express themselves and therefore can better regulate their emotions, which may avoid the development of internalizing and externalizing symptoms [46]. The association of language ability with child behavior was evident only from ages 7 to 11. Associations at earlier ages found in the CLPM became non-significant in the RI-CLPM, suggesting that the early onset and co-occurring nature of language ability and internalizing and externalizing symptoms is important to take into account. Potentially, this finding points at a shared causal mechanism underlying these relationships at younger ages. We hypothesize that at older ages, peer relationships and academic demands become more complex. If a child is not able to adapt to these more complex situations, emotional and behavioral problems may arise. However, these causal associations remain to be evaluated.

Some limitations of our study must be discussed. First, internalizing and externalizing symptoms were mostly assessed by parents. Parents and teachers often agree on child externalizing symptoms [47], although parents generally have a better view of child internalizing symptoms than teachers [48]. Yet, the sensitivity analysis with the parent assessment at age 7 replaced by the teacher assessment yielded similar results. Second, internalizing symptoms might best be reported by children themselves [48], as parents may underreport problems which could have led to an underestimation of the association between internalizing symptoms and language ability. Even if this were the case, for young children, care service use is triggered by parents’ or teachers’ perceptions of the child. Therefore, knowing how parental or teacher report of internalizing symptoms is associated with language ability is useful for designing appropriate screening strategies. Another limitation of our study is that language ability at age 7 measured a slightly different construct to the assessments at ages 3, 5 and 11. Nonetheless, all assessments were correlated with similar effect sizes and have been shown to be related cross-sectionally and longitudinally [23, 24], supporting the shared language construct of expressive vocabulary. Fourth, the time intervals between ages three and five, and between ages five and seven were similar, but the last time interval, between ages seven and 11, was longer. As these time intervals were not explicitly modeled in RI-CLPM, bias might have been introduced by the unequal time intervals between assessments.

A notable strength of this study is the use of an analytic technique to model the longitudinal associations after controlling for trait levels and prior states, which is important to disentangle early concurrent associations between internalizing symptoms, externalizing symptoms and poor language ability and the risk of developing another condition due to a pre-existing one. Additionally, the present study covered preschool and primary school periods, which are marked by rapid child development. Because internalizing symptoms, externalizing symptoms and poor language ability have their origin early in life, population-based studies from a young age onwards are needed, as most research focuses on children at high risk of developing disorders or clinical cases, who already have problems.

In future research, extensive, consistent and repeated assessments of language ability are required to appropriately assess the mechanisms linking behavior, emotions and language development. Besides, the study of gene-environment relations is expanding, with a substantial consensus of the role of the environment in the occurrence of genetically predisposed disorders. Beyond considering genetic endowment as a confounder, genetic factors should be evaluated as interactors or as additional exposures that confer risk of disease and environmental factors as their mediators.

Results from our analysis suggest that internalizing symptoms, externalizing symptoms and language difficulties start to arise early in life and co-occur throughout childhood. Clearly, health care professionals should be aware of this co-occurrence, that if a child presents symptoms in one domain, it is important to screen for problems in each domain. Additionally, in early childhood, children with externalizing symptoms might be at risk of delayed language acquisition and of developing internalizing symptoms. In early primary school, children with poorer language ability are at risk of developing internalizing and externalizing symptoms. Although reflecting small mean effect sizes, children with poor language ability may benefit from monitoring of their behavior and emotions. Whether the development of behavioral and emotional problems can be prevented, should be tested in an intervention study that focusses on improving language skills in early primary school.

### Supplementary Information

Below is the link to the electronic supplementary material.

Supplementary file1 (DOCX 204 KB)

## Data Availability

Researchers can access most of the MCS survey data through the University of Essex’s UK Data Service. Individuals who want to access the data must register with the UK Data Service prior to downloading it.
