# Supplementary material for: Bidirectional associations between mental health problems and language ability across 8 years of childhood
Source: Eur Child Adolesc Psychiatry. 2023 Apr 3;33(3):787–97. doi: 10.1007/s00787-023-02192-x (PMC10894104; doi:10.1007/s00787-023-02192-x)
Supplement: Supplementary file 1 — Supplementary file1 (DOCX 204 KB) [file 787_2023_2192_MOESM1_ESM.docx]

**Supplementary material**

**Supplementary Table 1.** *Factor* structure *of internalizing and externalizing scales in the study population*

|  | CFIr | RMSEAr | SRMR |
| --- | --- | --- | --- |
| Internalizing age 3 | 0.95 | 0.04 | 0.02 |
| Externalizing age 3 | 0.97 | 0.05 | 0.03 |
| Internalizing age 5 | 0.96 | 0.04 | 0.02 |
| Externalizing age 5 | 0.97 | 0.05 | 0.02 |
| Internalizing age 7 mother rated | 0.93 | 0.05 | 0.04 |
| Externalizing age 7 mother rated | 0.97 | 0.06 | 0.03 |
| Internalizing age 7 teacher rated | 0.98 | 0.04 | 0.02 |
| Externalizing age 7 teacher rated | 0.98 | 0.07 | 0.03 |
| Internalizing age 11 | 0.99 | 0.04 | 0.02 |
| Externalizing age 11 | 0.97 | 0.08 | 0.04 |

CFIr= Robust Comparative Fit Index, RMSEAr= Robust Root Mean Square Error of Approximation, SRMR= Standardized Root Mean Square Residual (; acceptable fit ≤0.08)

**Supplementary Table 2.** *Summary of assessments*

|  | Child’s age years | Behavior and emotions reported by | Language ability assessment |
| --- | --- | --- | --- |
| Wave 2 | 3 | Mother | BAS Naming Vocabulary |
| Wave 3 | 5 | Mother | BAS Naming Vocabulary |
| Wave 4 | 7 | Mother and teacher | BAS Word Reading |
| Wave 5 | 11 | Teacher | BAS Verbal Similarities |

All behavior and emotions assessments correspond to the SDQ. BAS= British Ability Scales. All language ability were assessed by a trained interviewer.

**Supplementary Table 3.** *Correlation coefficients.*

|  | Int3 | Ext3 | L3 | Int5 | Ext5 | L5 | Int7 | Ext7 | L7 | Int11 | Ext11 | L11 |
| --- | --- | --- | --- | --- | --- | --- | --- | --- | --- | --- | --- | --- |
| Int3 | 1 | 0.39 | -0.19 | 0.49 | 0.29 | -0.16 | 0.43 | 0.27 | -0.16 | 0.16 | 0.13 | -0.18 |
| Ext3 |  | 1 | -0.24 | 0.32 | 0.61 | -0.21 | 0.32 | 0.56 | -0.25 | 0.2 | 0.29 | -0.21 |
| L3 |  |  | 1 | -0.18 | -0.24 | 0.51 | -0.17 | -0.23 | 0.33 | -0.15 | -0.22 | 0.31 |
| Int5 |  |  |  | 1 | 0.41 | -0.18 | 0.60 | 0.34 | -0.17 | 0.23 | 0.19 | -0.18 |
| Ext5 |  |  |  |  | 1 | -0.23 | 0.37 | 0.72 | -0.29 | 0.24 | 0.41 | -0.23 |
| L5 |  |  |  |  |  | 1 | -0.16 | -0.21 | 0.40 | -0.17 | -0.22 | 0.41 |
| Int7 |  |  |  |  |  |  | 1 | 0.46 | -0.2 | 0.29 | 0.23 | -0.18 |
| Ext7 |  |  |  |  |  |  |  | 1 | -0.32 | 0.28 | 0.47 | -0.23 |
| L7 |  |  |  |  |  |  |  |  | 1 | -0.22 | -0.32 | 0.35 |
| Int11 |  |  |  |  |  |  |  |  |  | 1 | 0.44 | -0.19 |
| Ext11 |  |  |  |  |  |  |  |  |  |  | 1 | -0.23 |
| L5 |  |  |  |  |  |  |  |  |  |  |  | 1 |

Prefixes: Int, Ext and L: Internalizing symptoms, externalizing symptoms and language assessments. Suffixes 3, 5, 7 and 11: represent age at assessment. Pearson’s correlation coefficients are presented.

**Supplementary Table 4.** *Bidirectional association of internalizing and externalizing problems and language ability.*

|  |  | Model 1 RI-CLPM | | |  | Model 2 CLPM |  |
| --- | --- | --- | --- | --- | --- | --- | --- |
|  |  | β | *95% CI* | *p* | β | *95% CI* | *p* |
| Child age 3 years | Child age 5 years |  |  |  |  |  |  |
| Internalizing | Internalizing | 0.31 | (0.27, 0.35) | <.001 | 0.42 | (0.40, 0.44) | <.001 |
| Externalizing | Internalizing | 0.13 | (0.10, 0.16) | <.001 | 0.06 | (0.05, 0.08) | <.001 |
| Language | Internalizing | -0.002 | (-0.04, 0.03) | 0.94 | -0.05 | (-0.07, -0.02) | <.001 |
| Internalizing | Externalizing | 0.04 | (0.001, 0.08) | 0.04 | 0.05 | (0.02, 0.08) | <.001 |
| Externalizing | Externalizing | 0.48 | (0.43, 0.51) | <.001 | 0.45 | (0.43, 0.47) | <.001 |
| Language | Externalizing | -0.02 | (-0.05, 0.03) | 0.54 | -0.11 | (-0.14, -0.08) | <.001 |
| Internalizing | Language | 0.05 | (0.02, 0.07) | 0.002 | -0.01 | (-0.02, 0.01) | 0.29 |
| Externalizing | Language | -0.01 | (-0.04, 0.002) | 0.07 | -0.03 | (-0.04, -0.02) | <.001 |
| Language | Language | 0.26 | (0.22, 0.28) | <.001 | 0.45 | (0.42, 0.46) | <.001 |
| Child age 5 years | Child age 7 years |  |  |  |  |  |  |
| Internalizing | Internalizing | 0.45 | (0.41, 0.49) | <.001 | 0.60 | (0.57, 0.62) | <.001 |
| Externalizing | Internalizing | 0.16 | (0.12, 0.18) | <.001 | 0.11 | (0.09, 0.12) | <.001 |
| Language | Internalizing | 0.02 | (-0.02, 0.05) | 0.38 | -0.01 | (-0.04, 0.01) | 0.36 |
| Internalizing | Externalizing | 0.06 | (0.02, 0.09) | 0.001 | 0.08 | (0.04, 0.10) | <.001 |
| Externalizing | Externalizing | 0.62 | (0.58, 0.65) | <.001 | 0.66 | (0.64, 0.68) | <.001 |
| Language | Externalizing | 0.02 | (-0.03, 0.05) | 0.59 | -0.02 | (-0.06, 0.00) | 0.05 |
| Internalizing | Language | 0.01 | (-0.02, 0.04) | 0.54 | -0.02 | (-0.05, 0.01) | 0.20 |
| Externalizing | Language | -0.14 | (-0.17, -0.12) | <.001 | -0.17 | (-0.20, -0.16) | <.001 |
| Language | Language | 0.19 | (0.14, 0.23) | <.001 | 0.52 | (0.48, 0.55) | <.001 |
| Child age 7 years | Child age 11 years |  |  |  |  |  |  |
| Internalizing | Internalizing | 0.11 | (0.05, 0.13) | <.001 | 0.22 | (0.18, 0.25) | <.001 |
| Externalizing | Internalizing | 0.12 | (0.09, 0.17) | <.001 | 0.09 | (0.07, 0.12) | <.001 |
| Language | Internalizing | -0.08 | (-0.12, -0.06) | <.001 | -0.10 | (-0.13, -0.08) | <.001 |
| Internalizing | Externalizing | 0.004 | (-0.03, 0.06) | 0.58 | -0.004 | (-0.04, 0.04) | 0.96 |
| Externalizing | Externalizing | 0.29 | (0.21, 0.32) | <.001 | 0.33 | (0.29, 0.35) | <.001 |
| Language | Externalizing | -0.13 | (-0.17, -0.10) | <.001 | -0.18 | (-0.21, -0.15) | <.001 |
| Internalizing | Language | -0.01 | (-0.05, 0.01) | 0.27 | -0.03 | (-0.05, -0.02) | <.001 |
| Externalizing | Language | -0.02 | (-0.04, 0.02) | 0.62 | -0.03 | (-0.04, -0.02) | <.001 |
| Language | Language | 0.12 | (0.10, 0.14) | <.001 | 0.14 | (0.13, 0.15) | <.001 |
|  |  |  |  |  |  |  |  |
| CFIr |  | 0.94 |  |  | 0.91 |  |  |
| RMSEAr |  | 0.06 |  |  | 0.07 |  |  |
| SRMR |  | 0.04 |  |  | 0.05 |  |  |

Model 1 output corresponds to the RI-CLPM’s auto-regressive and cross-lagged paths depicted in Figure 1. Model 2 output corresponds to the CLPM’s auto-regressive paths depicted in Figure 1. CFIr: Robust comparative fit index. RMSEAr: Robust root mean square error of approximation. SRMR: Robust standardized root mean square residual.

**Supplementary Table 5.** *Bidirectional association of internalizing and externalizing problems and language ability for boys and girls.*

|  |  |  | Model 1 |  |  | Model 3 Boys |  |  | Model 4 Girls |  |
| --- | --- | --- | --- | --- | --- | --- | --- | --- | --- | --- |
|  |  | β | *95% CI* | *p* | β | *95% CI* | *p* | β | *95% CI* | *p* |
| Child age 3 years | Child age 5 years |  |  |  |  |  |  |  |  |  |
| Internalizing | Internalizing | 0.31 | (0.27, 0.35) | <.001 | 0.30 | (0.24, 0.35) | <.001 | 0.33 | (0.27, 0.38) | <.001 |
| Externalizing | Internalizing | 0.13 | (0.10, 0.16) | <.001 | 0.12 | (0.10, 0.18) | <.001 | 0.12 | (0.08, 0.15) | <.001 |
| Language | Internalizing | -0.002 | (-0.04, 0.03) | 0.94 | -0.01 | (-0.06, 0.04) | 0.70 | 0.005 | (-0.04, 0.05) | 0.94 |
| Internalizing | Externalizing | 0.04 | (0.001, 0.08) | 0.04 | 0.03 | (-0.02, 0.09) | 0.25 | 0.06 | (-0.002, 0.10) | 0.05 |
| Externalizing | Externalizing | 0.48 | (0.43, 0.51) | <.001 | 0.44 | (0.39, 0.50) | <.001 | 0.50 | (0.44, 0.52) | <.001 |
| Language | Externalizing | -0.02 | (-0.05, 0.03) | 0.54 | -0.02 | (-0.08, 0.04) | 0.51 | -0.03 | (-0.08, 0.03) | 0.40 |
| Internalizing | Language | 0.05 | (0.02, 0.07) | 0.002 | 0.05 | (0.01, 0.08) | 0.02 | 0.04 | (-0.002, 0.07) | 0.06 |
| Externalizing | Language | -0.01 | (-0.04, 0.002) | 0.07 | -0.001 | (-0.04, 0.02) | 0.46 | -0.03 | (-0.06, -0.01) | 0.01 |
| Language | Language | 0.26 | (0.22, 0.28) | <.001 | 0.24 | (0.19, 0.28) | <.001 | 0.28 | (0.24, 0.31) | <.001 |
| Child age 5 years | Child age 7 years |  |  |  |  |  |  |  |  |  |
| Internalizing | Internalizing | 0.45 | (0.41, 0.49) | <.001 | 0.46 | (0.41, 0.52) | <.001 | 0.45 | (0.38, 0.50) | <.001 |
| Externalizing | Internalizing | 0.16 | (0.12, 0.18) | <.001 | 0.14 | (0.10, 0.18) | <.001 | 0.17 | (0.12, 0.19) | <.001 |
| Language | Internalizing | 0.02 | (-0.02, 0.05) | 0.38 | 0.03 | (-0.04, 0.07) | 0.54 | 0.01 | (-0.04, 0.07) | 0.54 |
| Internalizing | Externalizing | 0.06 | (0.02, 0.09) | 0.001 | 0.07 | (0.03, 0.12) | 0.002 | 0.05 | (-0.004, 0.08) | 0.08 |
| Externalizing | Externalizing | 0.62 | (0.58, 0.65) | <.001 | 0.60 | (0.54, 0.64) | <.001 | 0.64 | (0.59, 0.67) | <.001 |
| Language | Externalizing | 0.02 | (-0.03, 0.05) | 0.59 | 0.02 | (-0.05, 0.07) | 0.66 | 0.01 | (-0.05, 0.06) | 0.97 |
| Internalizing | Language | 0.01 | (-0.02, 0.04) | 0.54 | 0.01 | (-0.04, 0.06) | 0.65 | 0.01 | (-0.04, 0.05) | 0.70 |
| Externalizing | Language | -0.14 | (-0.17, -0.12) | <.001 | -0.14 | (-0.20, -0.12) | <.001 | -0.16 | (-0.18, -0.12) | <.001 |
| Language | Language | 0.19 | (0.14, 0.23) | <.001 | 0.19 | (0.11, 0.24) | <.001 | 0.19 | (0.13, 0.26) | <.001 |
| Child age 7 years | Child age 11 years |  |  |  |  |  |  |  |  |  |
| Internalizing | Internalizing | 0.11 | (0.05, 0.13) | <.001 | 0.12 | (0.05, 0.17) | <.001 | 0.09 | (0.01, 0.13) | 0.02 |
| Externalizing | Internalizing | 0.12 | (0.09, 0.17) | <.001 | 0.11 | (0.08, 0.18) | <.001 | 0.13 | (0.08, 0.17) | <.001 |
| Language | Internalizing | -0.08 | (-0.12, -0.06) | <.001 | -0.07 | (-0.12, -0.05) | <.001 | -0.10 | (-0.14, -0.06) | <.001 |
| Internalizing | Externalizing | 0.004 | (-0.03, 0.06) | 0.58 | 0.002 | (-0.05, 0.08) | 0.60 | 0.02 | (-0.04, 0.06) | 0.69 |
| Externalizing | Externalizing | 0.29 | (0.21, 0.32) | <.001 | 0.27 | (0.18, 0.33) | <.001 | 0.29 | (0.20, 0.33) | <.001 |
| Language | Externalizing | -0.13 | (-0.17, -0.10) | <.001 | -0.14 | (-0.18, -0.10) | <.001 | -0.11 | (-0.16, -0.08) | <.001 |
| Internalizing | Language | -0.01 | (-0.05, 0.01) | 0.27 | 0.02 | (-0.04, 0.05) | 0.80 | -0.03 | (-0.08, -0.002) | 0.04 |
| Externalizing | Language | -0.02 | (-0.04, 0.02) | 0.62 | -0.03 | (-0.06, 0.02) | 0.36 | -0.02 | (-0.05, 0.02) | 0.45 |
| Language | Language | 0.12 | (0.10, 0.14) | <.001 | 0.14 | (0.12, 0.16) | <.001 | 0.11 | (0.09, 0.15) | <.001 |
|  |  |  |  |  |  |  |  |  |  |  |
| CFIr |  | 0.94 |  |  | 0.96 |  |  | 0.94 |  |  |
| RMSEAr |  | 0.06 |  |  | 0.05 |  |  | 0.06 |  |  |
| SRMR |  | 0.04 |  |  | 0.03 |  |  | 0.04 |  |  |

Model 3 N= 7,390. Model 4 N= 7,073. The models output correspond to the RI-CLPM’s auto-regressive and cross-lagged paths. Model 1 output corresponds to the RI-CLPM’s auto-regressive and cross-lagged paths depicted in Figure 1. Model 3 and 4 represent a similar analysis to model 1 depicted in Figure 1 further stratified for boys and girls respectively. CFIr: Robust comparative fit index. RMSEAr: Robust root mean square error of approximation. SRMR: Robust standardized root mean square residual.

**Supplementary Table 6.** *Bidirectional association of internalizing and externalizing problems and language ability, sensitivity analysis models.*

|  |  |  | Model 5 |  |  | Model 6 |  |
| --- | --- | --- | --- | --- | --- | --- | --- |
|  |  | β | *95% CI* | *p* | β | *95% CI* | *p* |
| Child age 3 years | Child age 5 years |  |  |  |  |  |  |
| Internalizing | Internalizing | 0.32 | (0.28, 0.35) | <.001 | 0.33 | (0.30, 0.36) | <.001 |
| Externalizing | Internalizing | 0.12 | (0.10, 0.15) | <.001 | 0.11 | (0.08, 0.14) | <.001 |
| Language | Internalizing | 0.01 | (-0.03, 0.04) | 0.74 | -0.002 | (-0.03, 0.03) | 0.98 |
| Internalizing | Externalizing | 0.04 | (0.0001, 0.08) | 0.04 | 0.02 | (-0.01, 0.06) | 0.19 |
| Externalizing | Externalizing | 0.47 | (0.43, 0.50) | <.001 | 0.47 | (0.44, 0.50) | <.001 |
| Language | Externalizing | -0.001 | (-0.04, 0.04) | 0.90 | -0.01 | (-0.05, 0.03) | 0.78 |
| Internalizing | Language | 0.06 | (0.03, 0.07) | <.001 | 0.04 | (0.01, 0.06) | 0.001 |
| Externalizing | Language | 0.02 | (-0.004, 0.03) | 0.14 | -0.01 | (-0.03, 0) | 0.06 |
| Language | Language | 0.22 | (0.19, 0.24) | <.001 | 0.26 | (0.23, 0.28) | <.001 |
| Child age 5 years | Child age 7 years |  |  |  |  |  |  |
| Internalizing | Internalizing | 0.46 | (0.41, 0.50) | <.001 | 0.06 | (0.02, 0.13) | 0.01 |
| Externalizing | Internalizing | 0.15 | (0.12, 0.17) | <.001 | 0.08 | (0.05, 0.13) | <.001 |
| Language | Internalizing | 0.03 | (-0.01, 0.06) | 0.17 | -0.01 | (-0.07, 0.03) | 0.54 |
| Internalizing | Externalizing | 0.05 | (0.02, 0.09) | 0.001 | -0.01 | (-0.06, 0.04) | 0.66 |
| Externalizing | Externalizing | 0.61 | (0.57, 0.64) | <.001 | 0.24 | (0.20, 0.29) | <.001 |
| Language | Externalizing | 0.04 | (-0.01, 0.07) | 0.17 | -0.05 | (-0.09, 0.02) | 0.19 |
| Internalizing | Language | 0.02 | (-0.01, 0.05) | 0.23 | 0.002 | (-0.03, 0.04) | 0.72 |
| Externalizing | Language | -0.12 | (-0.16, -0.10) | <.001 | -0.14 | (-0.17, -0.12) | <.001 |
| Language | Language | 0.13 | (0.08, 0.17) | <.001 | 0.19 | (0.14, 0.23) | <.001 |
| Child age 7 years | Child age 11 years |  |  |  |  |  |  |
| Internalizing | Internalizing | 0.11 | (0.05, 0.13) | <.001 | 0.24 | (0.20, 0.28) | <.001 |
| Externalizing | Internalizing | 0.11 | (0.09, 0.16) | <.001 | 0.17 | (0.12, 0.20) | <.001 |
| Language | Internalizing | -0.07 | (-0.11, -0.05) | <.001 | -0.05 | (-0.09, -0.04) | <.001 |
| Internalizing | Externalizing | 0.01 | (-0.03, 0.06) | 0.58 | 0.01 | (-0.02, 0.04) | 0.39 |
| Externalizing | Externalizing | 0.28 | (0.21, 0.32) | <.001 | 0.56 | (0.51, 0.59) | <.001 |
| Language | Externalizing | -0.11 | (-0.15, -0.09) | <.001 | -0.06 | (-0.09, -0.03) | <.001 |
| Internalizing | Language | -0.01 | (-0.05, 0.01) | 0.24 | 0.02 | (-0.03, 0.03) | 0.92 |
| Externalizing | Language | -0.01 | (-0.02, 0.03) | 0.90 | -0.01 | (-0.02, 0.02) | 0.67 |
| Language | Language | 0.08 | (0.06, 0.10) | <.001 | 0.12 | (0.11, 0.14) | <.001 |
|  |  |  |  |  |  |  |  |
| CFIr |  | 0.93 |  |  | 0.94 |  |  |
| RMSEAr |  | 0.06 |  |  | 0.05 |  |  |
| SRMR |  | 0.04 |  |  | 0.04 |  |  |

The models output correspond to the RI-CLPM’s auto-regressive and cross-lagged paths. Model 5 assessed the bidirectional association of internalizing problems, externalizing problems and language ability as model 1 further adjusting for child IQ. Model 6 assessed the bidirectional association of internalizing problems, externalizing problems and language ability as model 1 with the SDQ teacher assessment at age 7. Significant paths are presented in Supplementary Figure 2. CFIr: Robust comparative fit index. RMSEAr: Robust root mean square error of approximation. SRMR: Robust standardized root mean square residual.

**Supplementary Figure 1.** *Bidirectional association of internalizing and externalizing problems and language ability.*


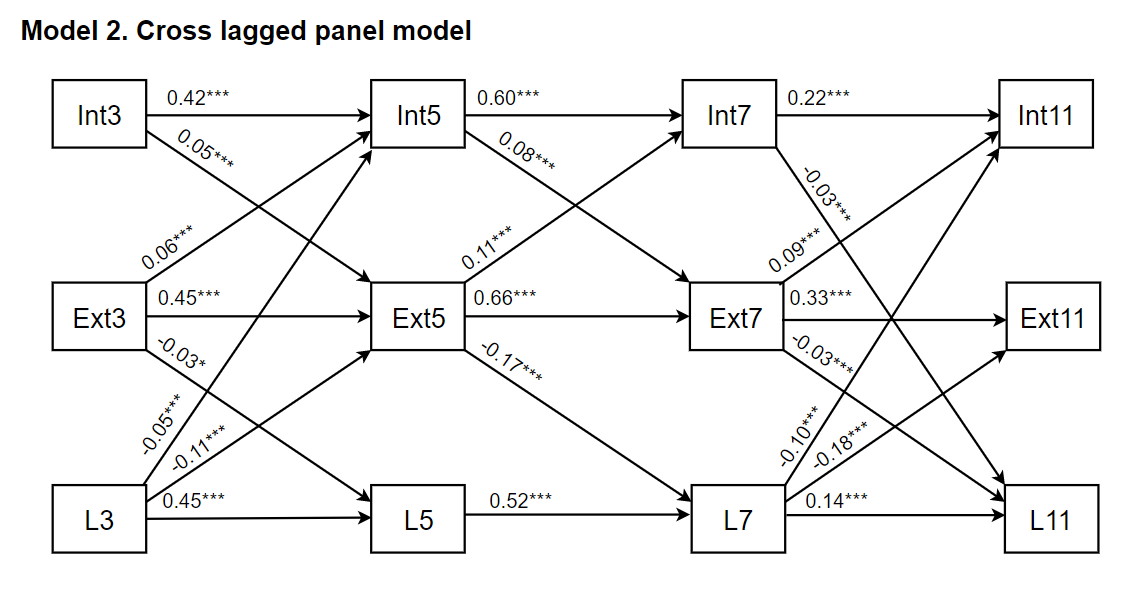


The output corresponds to the CLPM’s auto-regressive and cross-lagged paths depicted as Model 2 in Supplementary figure 1. Prefixes L, Ext and Int: Language assessments, externalizing and internalizing problems. Suffixes 3, 5, 7 and 11: represent age at assessment. The complete output of all these paths is presented in Supplementary Table 1.

**Supplementary Figure 2.** *Bidirectional association of internalizing and externalizing problems and language ability, sensitivity analysis models.*


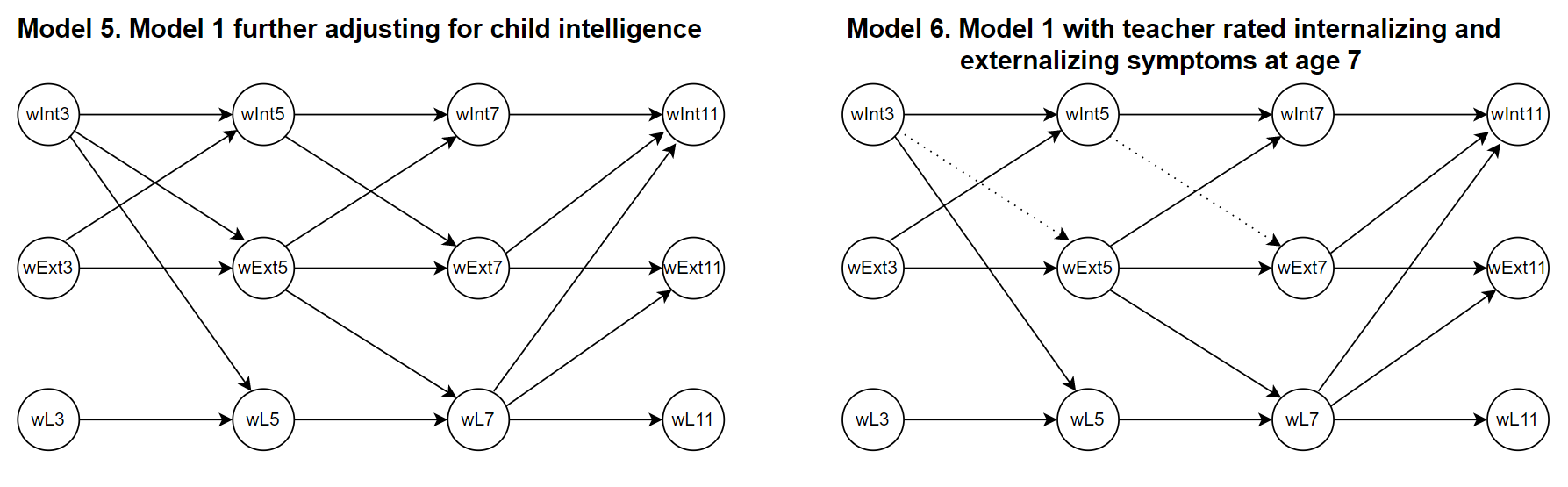


The models output correspond to the RI-CLPM’s auto-regressive and cross-lagged paths. Model 5 assessed the bidirectional association of internalizing problems, externalizing problems and language ability as model 1 further adjusting for child IQ. Model 6 assessed the bidirectional association of internalizing problems, externalizing problems and language ability as model 1 with the SDQ teacher assessment at age 7. Complete output of these paths is presented in Supplementary Table 4.
